# A prospective cohort study on longitudinal trajectories of cognitive function after stroke

**DOI:** 10.1038/s41598-021-96347-y

**Published:** 2021-08-26

**Authors:** Dongni Buvarp, Lena Rafsten, Tamar Abzhandadze, Katharina S. Sunnerhagen

**Affiliations:** 1 Rehabilitation Medicine Research Group, Department of Clinical Neuroscience, Institute of Neuroscience and Physiology, University of Gothenburg, Gothenburg, Sweden; 2 Sahlgrenska University Hospital, Gothenburg, Sweden

**Keywords:** Stroke, Outcomes research

## Abstract

The study aimed to determine longitudinal trajectories of cognitive function during the first year after stroke. The Montreal Cognitive Assessment (MoCA) was used to screen cognitive function at 36–48 h, 3-months, and 12-months post-stroke. Individuals who shared similar trajectories were classified by applying the group-based trajectory models. Data from 94 patients were included in the analysis. Three cognitive functioning groups were identified by the trajectory models: high [14 patients (15%)], medium [58 (62%)] and low [22 (23%)]. For the high and medium groups, cognitive function improved at 12 months, but this did not occur in the low group. After age, sex and education matching to the normative MoCA from the Swedish population, 52 patients (55%) were found to be cognitively impaired at baseline, and few patients had recovered at 12 months. The impact on memory differs between cognitive functioning groups, whereas the impact on activities of daily living was not different. Patients with the poorest cognitive function did not improve at one-year poststroke and were prone to severe memory problems. These findings may help to increase focus on long-term rehabilitation plans for those patients, and more accurately assess their needs and difficulties experienced in daily living.

## Introduction

Cognitive impairment is a highly prevalent long-term stroke consequence, with approximately 80% of patients reporting mild cognitive impairments post-stroke^1–4^. In addition to motor impairments, cognition decline is one of the key determinants of deterioration in performance of daily activities after stroke^5^. The ability to early recognize individuals with potential cognitive decline after stroke would make it possible to appropriately assess their actual needs and additional supports, while tailoring interventions towards these individuals, thus improving their quality of life.

Longitudinal changes in cognitive function after stroke were largely diverse. Multiple evolving trends, such as improvement, worsening, or remaining stable, are shown to depend on the use of cognitive instruments, as well as the length of time until follow-up, whether short-term or long-term^6^. A significant decline in global cognition was demonstrated a few years after stroke (from 3 to 6 years)^7,8^, whereas significant improvements in cognition were found within 6-months post-stroke^9,10^. Higher age and post-stroke depression in individuals were associated with a cognitive decline^11,12^. Mixed findings were reported for other risk factors (e.g. sex, ApoE ε4 status, vascular risk factors)^6,13^. However, the risk of post-stroke dementia may likely be related to the incidence of stroke rather than to the background vascular risk factors^1^. To date, it remains unclear how the longitudinal progress of post-stroke cognition varies in patients with different degrees of cognitive functioning in the acute phase. It may be that individuals with baseline severe cognitive deficits are more likely to suffer a cognitive decline. But if so, it is also not clear when this cognitive decline will occur. Moreover, it is also unknown if patients with varying baseline degrees of cognition will share similar trajectories over time. Exploring these longitudinal trajectories is, therefore, warranted to enhance understanding of the recovery of cognitive function post-stroke while deciphering the heterogeneity^14^. This knowledge will assist in constructing an early individual rehabilitation plan.

The study aimed to identify trajectories of cognition during the first year after stroke, and to examine the impact of different trajectory groups in relation to memory performance and activities of daily living.

## Method

### Data and material availability

The complete dataset cannot be made publicly available for ethical and legal reasons, according to Swedish regulations (https://etikprovning.se/for-forskare/ansvar/). Researchers can request access to the data by emailing the principal investigator at ks.sunnerhagen@neuro.gu.se.

### Study sample and design

This longitudinal study was approved by the Regional Ethical Review Board in Gothenburg (registration number: 426-05 and 042-11) and was conducted in agreement with the Declaration of Helsinki. Data was used from the Gothenburg Very Early Supported Discharge clinical trial (URL: http://www.clinicaltrials.gov. Unique identifier: NCT01622205) at the stroke unit of the Sahlgrenska University Hospital, Sweden, from September 2011 to April 2016^15^. The study participants were required to understand the study protocol and written informed consent was provided prior to the clinical trial. The inclusion criteria were age > 18 years, a diagnosis of stroke according to World Health Organization criteria^16^, a National Institute of Health Stroke Scale (NIHSS) score from 0 to 16 at Day 2, a Barthel Index (BI) from 50 to 100, and a score of less than 26 in The Montreal Cognitive Assessment (MoCA) if the BI score was 100. Exclusion criteria were if the participants were living in a nursing facility prior to stroke, lived more than 30 min traveling time to the Sahlgrenska University Hospital, were not able to communicate in Swedish, NIHSS > 16 and life expectancy less than one year (e.g. malign disease condition). Other inclusion and exclusion criteria of the clinical trial were previously reported^15^.

### Clinical assessments

The MoCA was administrated to evaluate cognitive functioning at an interval of 36 to 48 h after stroke (referred to as baseline), 3-months and 12-months after stroke. The MoCA is a valid and reliable instrument for screening of global cognition in patients with mild to moderate stroke^17,18^. The MoCA consists of subdomains following visuospatial/executive, naming, attention, abstraction, delayed recall or memory and orientation. A maximum score is 30, where lower indicates worse cognition^19^. One extra point was added to the final score if patients had less than or equal to 12 years of education. Written permission for using the MoCA test was obtained from MoCA© (MoCA—Cognitive Assessment https://www.mocatest.org).

Neurological deficits were evaluated using NIHSS at Day 2 after admission^20^. Motor-sensory function in the extremities was tested using the Fugl-Meyer Assessment Scale (FMA; lower extremity [-LE] and upper extremity [-UE]), with a lower FMA score indicating a more severe impairment of function^21^. Clinical measurements were conducted by an experienced physiotherapist and/or occupational therapist.

### Self-assessed measures

Psychological distress was assessed using the 14-item Hospital Anxiety and Depression Scale (HADS) self-assessment questionnaire, which comprised a 7-item subscale to assess anxiety and a 7-item subscale to assess depression^22^. Each item consists of four response levels, scored from 0 to 3. Mild and moderate anxiety or depression is attributed to a score above 7 points on the subscale^22^.

Memory performance and activities of daily living, two major domains which impact health-related quality of life after stroke, were assessed using the Stroke Impact Scale (SIS, version 3.0)^23^. The SIS is a 59-item multi-dimensional self-assessed measure of eight domains: strength, memory and thinking, emotion, communication, activities of daily living, mobility, hand function, and participation. Each domain contains a various number of items and each item has five response level to score their self-perceived difficulties after stroke. Memory (8 items) and ADL/IADL (10 items) of SIS-subdomains were used in this study to determine the impact of cognitive function in patients after stroke. Written permission of using the SIS scale version 3.0 was granted in 2006 from MAPI Research Trust, Lyon, France (https://www.mapi-trust.org). All the self-reported assessments were administered on Day 5 to not overburden the patients.

### Statistical analysis

#### Longitudinal trajectories of cognition

Cognition assessed by MoCA was transformed through converting each individual’s MoCA score into proportions, by dividing by the maximal MoCA score (30 points). The proportional MoCA scores are continuous with an interval from 0 to 1, and an upper and lower limited bound of 0.005 and 0.995 were used, respectively, to fit a beta distribution. Patients were considered lost to follow-up if there were more than two missed visits and were further excluded from the longitudinal analysis.

The longitudinal changes in cognition of the study sample were analyzed using a longitudinal beta regression mixed-effect model, while adjusting for age and sex^24^. Age was dichotomized to < 75 and ≥ 75 years using the median value. Time, age and sex were included as fixed effect, and random intercept was also used. Akaike information criterion, Bayesian information criterion, and pseudo-*R*^2^ were used to evaluate the model performance.

A group-based trajectory model was used for clustering, which is a finite mixture modelling designed to classify individuals who share similar longitudinal trajectories into clusters by fitting the developmental course of each individual over time using maximum likelihood estimation^25,26^. Group-based trajectory models specified for beta distribution were applied in this study to identify the subgroups that followed similar progression of cognition over time after stroke^27^. The subgroups were stratified through the highest predicted probability assigned to individuals. Model evaluation and selection for developmental trajectories was based on the Bayesian information criterion and average posterior probability.

### Cognitive impairments

By identifying patients with cognitive impairments, normative data on MoCA from the Swedish general population was used as a reference by matching age, sex and education^28^. Cognitive impairment was defined by whether the individuals’ total MoCA score was larger than or equal to 1 standard deviation of normative cognition after age-, sex-, and education-matching, after removal of an extra point for low education^28^. The number of patients with cognitive impairments in different trajectory groups was explored across the three time points.

### Impact of longitudinal cognition on activities of daily living and memory

Both memory and ADL/IADL domains of SIS were transformed to a continuous index, ranging from 0 to 100 (a lower score indicates a higher perceived impact)^29^. The means scores for SIS-memory performance and SIS-ADL/IADL were compared between different trajectory groups, as well as between different time points.

For group comparison, either Fisher’s exact test, Pearson’s χ^2^ test, Mann–Whitney *U* test, independent *t* tests, Kruskal–Wallis test, and 1-way analysis of variance was used, as appropriate. To compare changes between different time points in SIS and the subdomains of MoCA, either Friedman Test, repeated-measures analysis of variance, paired *t* test or Wilcoxon signed-rank was used, as appropriate. Significance for multiple comparisons was adjusted using Holm-Bonferroni corrections. A two tailed significance level was defined as *p* < 0.05. The effect size was also calculated for each applied statistical test to determine the magnitude of difference. Statistical analyses were performed using SAS (SAS Institute Inc., Cary, NC, USA) and IBM SPSS statistics 25 (IBM Corp., Armonk, NY).

## Results

**Figure 1:**
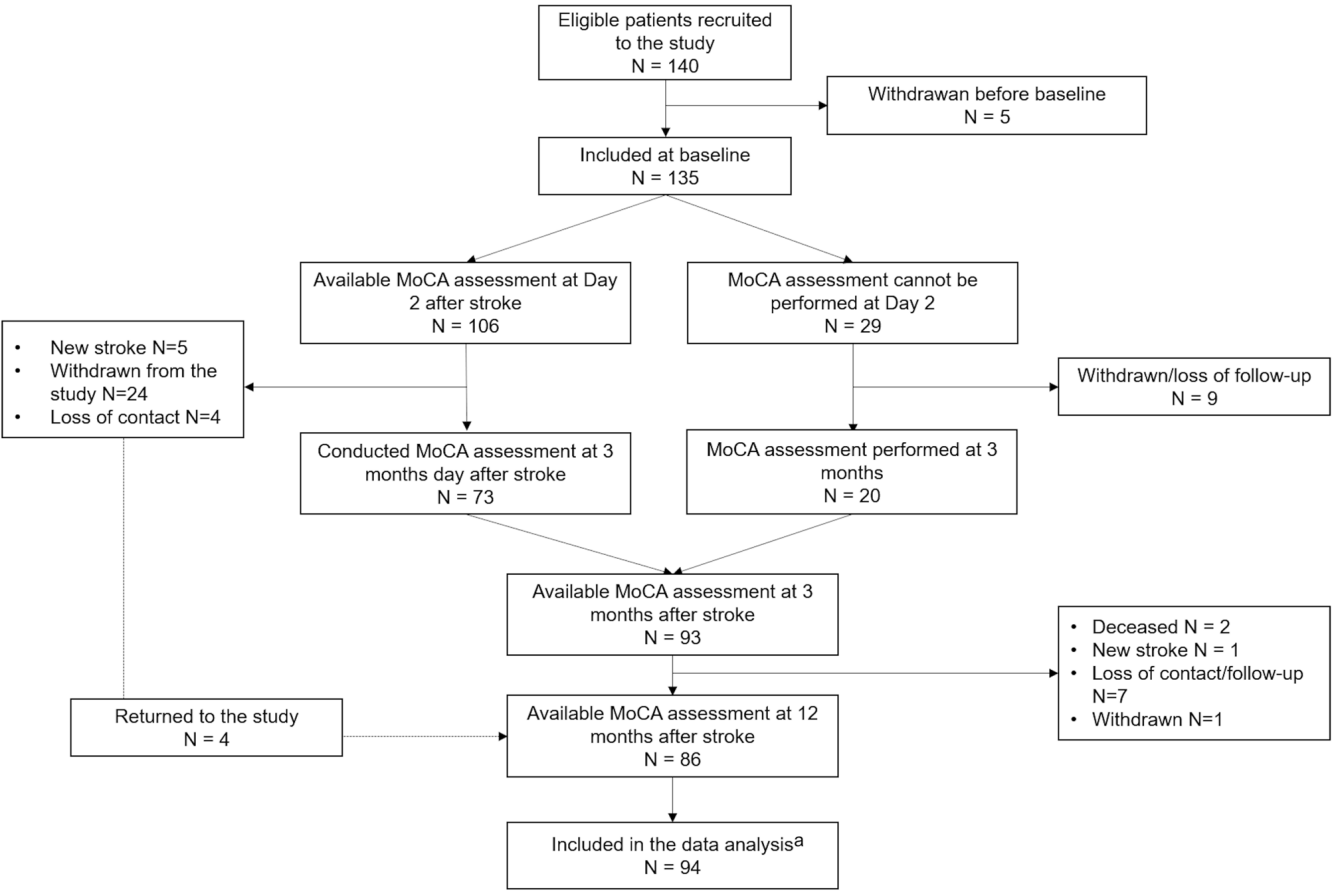
Flow chart summarizing study sample recruitment and inclusion and exclusion for longitudinal data analysis. ^a^94 patients who had two or more longitudinal assessments of MoCA were included into the analysis.

One hundred and forty patients were eligible for the study trial and five patients withdrew prior to the baseline assessments. Details of inclusion and exclusion in the longitudinal data analysis are presented in a flow chart (Fig. 1). No statistically significant difference was found between the patients excluded and those included in the study in terms of age, sex and neurological deficits. Data from 94 patients were included in the longitudinal analysis [median age 76 years, range 37–96, 40 females (43%); Table 1].

**Table 1:** Clinical assessments and self-reported measures between the identified trajectory groups.

Characteristic	All (N = 94)	High cognitive functioning-Cluster I (N = 14)	Medium cognitive functioning-Cluster II (N = 58)	Low cognitive functioning-Cluster III (N = 22)	*p*-value^a^
**Age [N (%)]**					**0.04**
< 75 years ≥ 75 years	41 (44) 53 (56)	9 (64) 5 (36)	27 (47) 31 (53)	5 (23) 17 (77)	
**Sex [N (%)]**					0.69
Male Female	53 (56) 41 (44)	7 (50) 7 (50)	32 (55) 26 (45)	14 (64) 8 (36)	
**Education [N (%)]**					0.12
Max 12 years > 12 years	34 (36) 60 (64)	2 (14) 12 (86)	25 (43) 33 (57)	7 (32) 15 (68)	
**Stroke type [N (%)]**					0.22
Ischemic infarct Intracerebral hemorrhage	88 (94) 6 (6)	14 (100) 0 (0)	55 (95) 3 (5)	19 (86) 3 (14)	
**Hemisphere of lesion [N (%)]**					0.35
Left Right Bilateral Cerebellum Brain stem Unclear	20 (21) 27 (29) 4 (4) 8 (9) 2 (2) 33 (35)	1 (7) 5 (36) 0 (0) 3 (21) 1 (7) 4 (29)	14 (24) 18 (31) 2 (3) 4 (7) 1 (2) 19 (33)	5 (23) 4 (18) 2 (9) 1 (5) 0 (0) 10 (45)	
**OCSP classification [N (%)]**^**b**^					0.31
TACS PACS LACS POCS Uncertain	3 (3) 14 (15) 34 (36) 30 (32) 7 (7)	0 (0) 3 (21) 4 (29) 6 (43) 1 (7)	2 (3) 6 (10) 23 (40) 21 (36) 3 (5)	1 (5) 5 (23) 7 (32) 3 (14) 3 (14)	
NIHSS	2 (0–4)	2 (1–7)	2 (0–4)	3 (2–5)	0.18
FMA-UE motor function	63 (57.8–65)	65 (63–66)	63 (57–64)	64 (58–65)	0.24
FMA-LE motor function	33 (28.5–34)	34 (29–34)	33 (29–34)	33 (30–34)	0.86
HADS-A	4 (1–7)	1 (0–3)	5 (1–6)	4 (0–9)	0.08
HADS-D	2.5 (1–6)	1 (0–1)	3 (1–6)	6 (2–7)	**0.002**
**MoCA, mean (SD)/median (IQR)**					
Baseline	21.9 (5)/22 (19–26)	26.8 (2)/27 (27–28)	22.5 (4)/23 (21–25)	16.6 (3)/16.5 (15–19)	** < 0.001**
3 months	23.4 (4)/24 (21–26)	28.4 (2)/28.5 (28–30)	24.5 (2)/25 (23–26)	16.8 (3)/16.5 (14.5–19)	** < 0.001**
12 months	23.8 (4)/25 (22–27)	28.5 (1)/29 (28–29)	24.8 (2)/25 (23–26)	17.2 (3)/18 (14–20)	** < 0.001**
***p*-value**^**c**^	** < 0.001**	**0.02**	**0.002**	0.37	
Changes from baseline to 3 months	1.8 (4)/1 (0–3)	1.5 (2)/2 (0–3)	2.3 (4)/1 (0–4)	0.86 (3)/1 (0–3)	
**Adjusted *p*-value**^**d**^	** < 0.001**	0.07	**0.002**	0.84	
Changes from 3 to 12 months	0.3 (2)/0 (−1 to 2)	−0.17 (2)/0 (−1.8 to 1)	0.6 (2)/0.5 (−1 to 2)	−0.6 (2)/−0.5 (−2.3 to 1.3)	
**Adjusted *p*-value**^**d**^	0.72	1.0	0.42	1.0	

Data are given as median (IQR) unless otherwise noted.

*IQR* interquartile range, *SD* standard deviation, *NIHSS* National Institutes of Health Stroke Scale (baseline N = 75), *MoCA* Montreal Cognitive Assessment (baseline N = 76), *FMA* Fugl-Meyer Assessment, *UE* upper extremity, *LE* lower extremity (baseline N = 93), *HADS* Hospital Anxiety and Depression Scale (baseline n = 134), *OCSP* Oxford Community Stroke Project classification, *TACS* total anterior circulation stroke, *PACS* partial anterior circulation stroke, *LACS* lacunar stroke, *POCS* posterior circulation stroke.

^a^Pearson’s χ^2^, Kruskal–Wallis test or 1-way analysis of variance were used as appropriate.

^b^Not applicable in 6 patients with intracerebral hemorrhage.

^c^Friedman test was used to explore changes in MoCA from baseline to 12 months after stroke.

^d^Wilcoxon signed-rank test was used, and multiple comparisons were adjusted using Holm-Bonferroni corrections.

### Longitudinal trajectories of cognitive function during the first year after stroke

Compared to baseline, there was a significant increase in MoCA scores of the whole study sample at 3 months [improved 2 scores; standardized β, 0.36 (95% CI, 0.2–0.5), adjusted p < 0.001], as well as at 12-months post-stroke [improved 3 scores; standardised β, 0.37 (95% CI, 0.22–0.52), adjusted p < 0.001], after adjustment for age and sex. No significant changes in cognitive scores were observed between 3- and 12-months post-stroke. Patients with age ≥ 75 years had poorer cognition over time [odds ratio (OR), 0.66 (95% CI, 0.46–0.93), p = 0.02]. Females showed a trend towards better cognition over time, however, this was not statistically significant [OR, 1.25 (95% CI 0.88–1.78), p = 0.2].

The cluster analysis using a group-based trajectory model identified three distinct groups: cluster I [14 out of 94 patients (15%)], cluster II [58 patients (62%)], and cluster III [22 patients (23%)]. The clinical characteristics of each clusters are shown in Table 1. Cluster I was characterised by high cognitive functioning, with the highest mean MoCA scores across the three time points. Cluster II was termed the medium cognitive functioning group, and had overall mean MoCA scores lower than cluster I, but higher than cluster III. The low cognitive functioning group (defined as cluster III) showed the lowest mean MoCA scores. There were significant differences between the three trajectory groups in total MoCA scores at baseline, 3-months, and 12-months post-stroke (Table 1).

**Figure 2:**
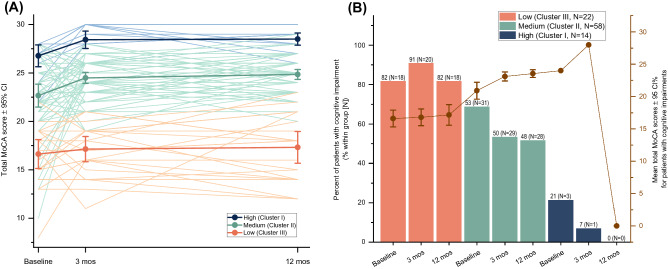
**(A)** Individual changes in total MoCA score over time in the different cognitive functioning groups (high, medium and low) and mean total MoCA scores of each cognitive functioning group are shown. **(B)** Percent of patients with cognitive impairment within each cognitive functioning group after age, sex and education-matching with the Swedish population. The light brown line represents the mean total MoCA of patients with cognitive impairment for each group. *MoCA* Montreal Cognitive Assessment, *CI* confidence interval, *mos* months.

For longitudinal changes in cognitive scores for each group, the high and medium cognitive functioning group showed significant improvements from baseline to 12 months (improved 2 median MoCA scores; p = 0.02, effect size Kendall’s W [W] = 0.32 for the high group; and p = 0.002, W = 0.17 for the medium group, Table 1). From baseline to 3-months, only the medium group showed significantly improved cognitive function (improved 1 median MoCA score, adjusted p = 0.002*,* effect size *r* = 0.54). No significant changes in cognition neither from baseline to 3 months, nor from 3- to 12-months post-stroke, were noted for the other two groups. Individual changes in cognitive functioning over time by the cognitive functioning groups are shown in Fig. 2.

**Figure 3:**
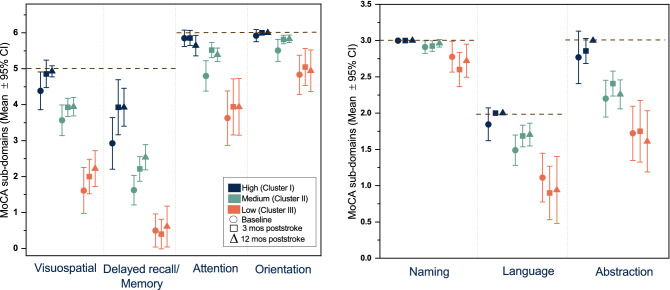
Mean scores of MoCA subdomains by different cognitive functioning groups over time. The black dash lines represent the maximum score of each subdomain. *MoCA* Montreal Cognitive Assessment, *CI* confidence interval, *mos* months.

The mean scores of MoCA subdomains by cognitive functioning groups over time are shown in Fig. 3. Most subdomains of MoCA showed statistically significant differences between cognitive functioning groups, except for the subdomain of naming at baseline.

For the high cognitive functioning group, a significant improvement in the memory domain from baseline to 12-months post-stroke was found (mean difference = 1.01, p = 0.049*,* W = 0.23). For the medium group, three subdomains were shown to be improved: attention (mean difference = 0.59, p = 0.001*,* W = 0.18), abstraction (mean difference = 0.21, p = 0.02*,* W = 0.1), and memory (mean difference = 0.92, p = 0.01*,* W = 0.12*).* For the low group, a significant decrease was found in language domain from baseline to 12 months (mean difference = − 0.11, p = 0.047, W = 0.26). No significant changes from baseline to 12 months were noted in any other subdomains of MoCA in the cognitive functioning groups.

### Cognitive impairments among different cognitive functioning groups

After matching for age, sex and education with the normative MoCA from the Swedish general population, 52 patients (55%) who were identified to have cognitive impairment at baseline, with 50 (53%), and 46 patients (49%) exhibiting impairment at 3 and 12 months, respectively. Among the 52 patients who had cognitive impairment at baseline, 32 (62%) remained cognitively impaired at 12-months post-stroke, while 13 patients (25%) recovered.

Among the cognitive functioning groups, there were significant differences in the number of patients with cognitive impairments at baseline, 3-months, and 12-months post-stroke (adjusted p < 0.001). The percentage of patients with cognitive impairments across the three time points in each group is presented in Fig. 1.

### Impact on memory performance and activities of daily living in different cognitive functioning groups

**Figure 4:**
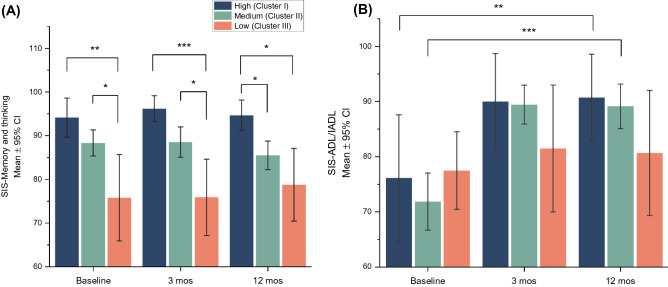
Impact on memory and activities of daily living by different cognitive functioning groups across 3 time points. Data available for SIS-memory and thinking in 93 patients at baseline, 87 patients at 3 and 12 months. Data available for SIS-ADL/IADL in 88 patients at baseline, 87 at 3 and 12 months. *p < 0.05, **p < 0.01, ***p < *0.001. SIS* stroke impact scale,* CI* confidence interval,* ADL* activities of daily living*, IADL* instrumental activities of daily living.

Among the cognitive functioning groups, there were significant differences between groups in memory performance, assessed by SIS, at baseline (adjusted p = 0.006, effect size ${E}_{R}^{2}$ = 0.14), 3-months (adjusted p = 0.003, ${E}_{R}^{2}$ = 0.18), and 12-months post-stroke (adjusted p = 0.01, ${E}_{R}^{2}$ = 0.13, Fig. 4). No significant differences between groups were found at any time points in the ADL/IADL domain assessed by SIS.

There was a significant change for the medium group in memory performance from baseline to 12-months post-stroke (mean difference = − 2.8, p = 0.01, W = 0.09). For the impact on ADL/IDL over time between groups, significant improvements were seen both for the medium and high group from baseline to 12-months post-stroke (mean difference = 14.6, p = 0.009*,* W = 0.36 for the high group*;* mean difference = 17.3, p < 0.001*,* W = 0.5 for the medium group). There were no significant improvements in SIS-ADL/IADL noted in the low group.

## Discussion

This longitudinal study demonstrated that the trajectories of cognition in stroke patients improved during the first three months, but subsequently remained unchanged until 12 months, after stroke. The group based-trajectory model stratified three levels of cognitive functioning: low, medium and high. After being age, sex, and education-matched with normative MoCA of the Swedish population, half of the study sample (55%) was found to have cognitive impairment at baseline, and 25% of these patients recovered after 12 months. The impact on memory performance significantly differed between cognitive functioning groups, whereas the impact of ALD/IADL was not different between groups.

Consistent with the general pattern of stroke recovery^30^, improvements in cognition were found solely within the first three months and tended to decline after that for the low cognitive functioning group in the present study. The improved cognition for the medium and high cognitive functioning groups was higher than the minimal clinically important difference of MoCA which with an estimate of 1.22 was previously suggested^31^. However, there were diverse patterns for changes in cognition shown after 3-months post-stroke or a longer time of follow-up, depending on the cognitive instruments used^10^. This may imply that the evolution of cognition is rather heterogenous and the rate of recovery differs significantly among stroke patients. The study findings based on the trajectory-model could contribute to deciphering heterogeneity by stratifying patients with similar developmental patterns over the course of time and identifying potential subgroups that may likely be susceptible to cognitive decline or poor recovery.

Patients with the poorest cognitive function, whose cognition did not improve during the first year after stroke, were older and had more frequent depressive symptom. This is in line with factors responsible for cognitive decline from earlier findings^11,12^. Furthermore, more severe initial cognitive impairment may reflect a decrease in neurological reserve and neuroplastic potential, which might lead to a poor recovery^32,33^. The low cognitive functioning group showed a substantially poor performance in the memory/delay recall domain over time and a significant decrease in language performance at 12-months post-stroke. The association between deficit of language expression and a greater risk of progressive cognitive decline was shown in a previous study^34^. Language deficits in stroke patients commonly involves left-side lesions, but this cannot be determined in our study sample due to the small sample size. However, various findings of the hemisphere of the lesion as a predictor for stroke recovery have previously been reported^35–37^.

Half of the study sample exhibited cognitive impairment at baseline. The prevalence in our study sample was similar in comparison with other studies that demonstrated a range from 30 to 74% depending on instruments used^3,4,38^. The patients who recovered at 12-months post-stroke were from the medium or high group, and none of the patients with cognitive impairment in the low group recovered. This further suggests that the patients with baseline severe cognitive deficits may show difficulty in recovering over time after stroke, and the loss of cognition may not be reversible.

Patients with lower cognitive functioning perceived a large impact on their memory performance as well as activities of daily living, compared to those with higher cognitive function. Perceived difficulties in these two SIS domains have been frequently reported in patients with cognitive impairment^39^. However, the perceived impact on memory performance did not change during the first year after stroke, and although there was a slight decrease in the mean score of the medium group, the effect size was considered to be very small. This could be due to a lack of awareness of cognitive problems. In contrast, the impact on activities of daily living was noted to be clinically meaningful, with changes from the baseline to 12-months post-stroke for the medium and the high group, as a clinically important difference of 5.9 was suggested^40^. The underlying reason maybe that the improved cognition has a positive impact on their activities of daily living, as was also previously shown^41^. Furthermore, as there was no group difference in ADL/IADL across any time points, this finding suggested that the patients with the poorest cognitive functioning may have difficulty to perceive their daily problems. This highlights the importance of recognising these patients early at baseline and the need to appropriately assess their potential difficulties along with a long-term follow-up plan.

The strength of the study is that the cognitive function assessed by MoCA was conducted in the very early stages of stroke and patients were followed up to 1 year. This captures a relatively complete picture of dynamic changes of cognition from the acute to chronic phase. The advance in the group-based trajectory model was able to identify patients with similar progress patterns, while deciphering certain heterogeneity from the disease group. Moreover, cognitive impairment was defined by age, sex and education-matching with the Swedish general population, which provides a more appropriate assessment of cognitive impairment in comparison to the use of a single cut-off of MoCA.

There were limitations that need to be addressed in this study. As one of the limitations, the included patients, in general, had mild to moderate neurological deficits; therefore, the cognitive function may be overestimated and thus have limited generalisability. However, it needs to be emphasised that a high score in ADL assessment does not necessarily suggest that patients do not suffer from cognitive impairment. With a mild cognitive impairment, patients may be able to perform basic activities of daily living, but there may be a more substantial impact on more complex activities that involve planning and executive function. This may explain why no group difference was found in SIS-ADL/IADL at any time point, as this domain was dominated by basic activities of living items. In addition, patients with severe cognitive deficits may have difficulties in realizing their limitations with basic ADL. The study findings may facilitate the improvement of recognition of cognitive impairments in those patients who score well in ADL assessments.

One more limitation is that the effect of some factors (such as localisation and initial clinical symptoms) on cognitive decline were not possible to explore due to the small sample size. Although substantial effort has been made to assess neuroimaging findings, precise lesions and volume were difficult to determine and were not within the scope of the study. This study has a primary interest in longitudinal trajectories in cognition assessed using MoCA. Furthermore, data on pre-morbid cognition as well as comorbidities were not available, and is also considered as one of the limitations. Including these variables into analyses, might offer a better understanding on longitudinal trajectories of cognition after stroke. The MoCA has similar limitations as in other cognitive instruments; for example, the ceiling effects on MoCA may be limited in its ability to detect potential improvement in the high cognitive functioning group^42^. The MoCA as a global measure of cognition may not capture subtle cognitive changes. There is a need for assessing executive function and subjective cognitive complaints, and tools for that are desirable. Also, the MoCA assessments could not be conducted for patients with aphasia or hemiplegia, which could result in an overestimation of the functioning. However, MoCA has previously been shown to be a feasible and sensitive tool for use in a very early stage of stroke to screen cognitive function and with excellent accuracy for detecting cognitive impairments among the available cognitive instruments^17,18^.

## Conclusion

Cognition after stroke, in general, improved during the first three months, but no significant improvement was found at any time point for patients with the poorest cognitive function who were older, more frequently suffering from depression, and prone to severe memory problems. This study may help to focus on rehabilitation plans for those patients and to more accurately assess their needs and difficulties in daily living.
